# To Petabytes and beyond: recent advances in probabilistic and signal processing algorithms and their application to metagenomics

**DOI:** 10.1093/nar/gkaa265

**Published:** 2020-04-27

**Authors:** R A Leo Elworth, Qi Wang, Pavan K Kota, C J Barberan, Benjamin Coleman, Advait Balaji, Gaurav Gupta, Richard G Baraniuk, Anshumali Shrivastava, Todd J Treangen

**Affiliations:** 1 Department of Computer Science, Houston, TX 77005, USA; 2 Systems, Synthetic, and Physical Biology (SSPB) Graduate Program, Houston, TX 77005, USA; 3 Department of Bioengineering, Houston, TX 77005, USA; 4 Department of Electrical and Computer Engineering, Rice University, Houston, TX 77005, USA

## Abstract

As computational biologists continue to be inundated by ever increasing amounts of metagenomic data, the need for data analysis approaches that keep up with the pace of sequence archives has remained a challenge. In recent years, the accelerated pace of genomic data availability has been accompanied by the application of a wide array of highly efficient approaches from other fields to the field of metagenomics. For instance, sketching algorithms such as MinHash have seen a rapid and widespread adoption. These techniques handle increasingly large datasets with minimal sacrifices in quality for tasks such as sequence similarity calculations. Here, we briefly review the fundamentals of the most impactful probabilistic and signal processing algorithms. We also highlight more recent advances to augment previous reviews in these areas that have taken a broader approach. We then explore the application of these techniques to metagenomics, discuss their pros and cons, and speculate on their future directions.

## INTRODUCTION

**Figure 1.**
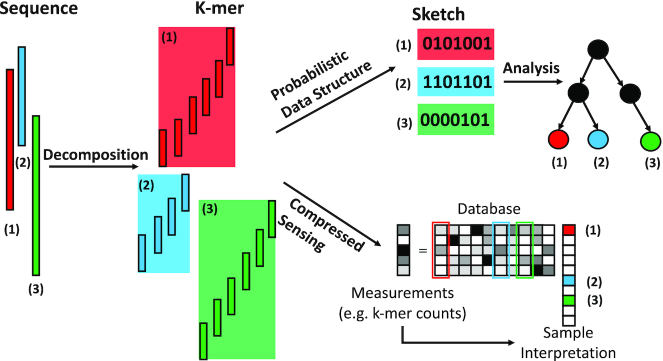
Overview of applying probabilistic data structures and compressed sensing in metagenomic sequence analysis. Given a set of sequences, each sequence is usually first decomposed into a series of consecutive k-mers. Then the probabilistic algorithm compresses the *k*-mers into sketches. The sketches can be analyzed to evaluate characteristics of the input sequences, such as sequence similarity. In compressed sensing (CS), the aggregate *k*-mer frequencies for the whole sample are treated as measurements. Elements of a database (e.g. microbial genomes) have individual *k*-mer frequency distributions that are stored in columns of a matrix. CS finds the elements of the database that comprise the sample measurements.

Thanks to advances in sequencing technology, the amount of next-generation sequencing data for genomics has increased at an exponential pace over the last decade. While this explosion of data has yielded unprecedented opportunities to answer previously unanswered questions in biology, it also creates new challenges. For instance, a key challenge is in designing new algorithms and data structures that are capable of handling analyses on such large and numerous datasets (Table 1). One approach for solving this big data problem is the development and adoption of probabilistic algorithms and data structures. When applying probabilistic methods to genomic analyses, input sequences are frequently decomposed into sets of overlapping subsequences with length *k*, referred to as *k*-mers. This large set of *k*-mers is then compressed into matrices using techniques from compressed sensing and sketching. Genomic analyses such as clustering and taxonomic classification can be performed directly on the compact matrices (Figure 1). In this paper, we review the great strides that have already been made in these areas and look forward to future possibilities.

**Table 1.** Terabyte and Petabyte scale datasets

Database	Size (PB)
Global Ocean Sampling (124)	<0.001
Ocean Sampling Day (125)	<0.001
MetaHit (126)	0.001
Tara Oceans (127)	0.007
Terragenome (128)	0.010
JGI IMG (129)	0.017
Human Microbiome Project (130)	0.043
The European Nucleotide Archive (ENA) (131)	0.379
NCBI Sequence Read Archive (132)	33.848

Many novel probabilistic and signal processing approaches for handling these massive amounts of genetic data have been previously reviewed (1–5). For instance, in (1) a comprehensive review was performed covering probabilistic algorithms and data structures such as MinHash (6) and Locality Sensitive Hashing (LSH) (7), Count-Min Sketch (CMS) (8), HyperLogLog (9) and Bloom filters (10). This review includes extensive details of how these data structures work, supporting theory behind each of them, as well as a brief discussion of their applications. However, the genomics applications for each approach were not thoroughly covered. Other more biologically motivated reviews include a review of compressive algorithms in (2) and sketching approaches in (3). In (2), techniques are covered such as the Burrows-Wheeler transform (BWT) (11), the FM-index (12), and other techniques based around exploiting redundancy in large datasets. A more in depth discussion of many of these topics can also be found in (3,4) includes a thorough review of compressed string indexes, LSH via sketches, CMS, Bloom filters, and minimizers (13), with accompanying applications in genomics for each.

While many techniques focus on efficient ways to represent a dataset, the compressed sensing (CS) technique from signal processing exploits the sparsity of signals for their efficient acquisition and interpretation. CS’s measurement efficiency often translates to significant reductions in cost and time. CS has previously found biomedical applications in microscopy (14) and rapid MRI acquisition (15). In this review, we summarize the essentials of CS, relate the technique to the other probabilistic data structures and algorithms, discuss relevant recent advances, and highlight corresponding applications in metagenomics. We direct interested readers to (16) for further discussion of the core concepts of CS and to the seminal works of (17) and (18) for more thorough analyses.

Most recently, a comprehensive review of sketching algorithms in genomics was performed in (5). This review covers approaches like MinHash, Bloom filters, CMS, HyperLogLog, the biological applications and implementations of each, and even includes a set of live, interactive notebooks with code examples of each approach. Given the wealth of previously performed reviews on these topics, we refer readers to the works above for more in depth explanations of these approaches along with their applications, implementations, and theory. Instead, we include only a brief review of these fundamental methodologies, followed by more recent advances in these areas, and finally their applications to metagenomics. Previous studies have often neglected more novel applications in metagenomic data given the new challenges it poses. Metagenome sequencing and analysis not only complicates established fundamental problems in comparative genomics but also adds entirely new problems. Therefore, we focus on how the aforementioned techniques can overcome unique hurdles in metagenomics.

## PROBABILISTIC ALGORITHMS AND DATA STRUCTURES

Recently, more attention has been given to the study of probabilistic algorithms (19) as a means to circumvent the widening gap between the explosion of data and our computing capabilities. Algorithms based on hashing and sketching (20–25) have been extensively used in the theoretical computer science and database literature for reducing the computations associated with processing massive web-scale datasets (26–30).

Hashing algorithms are typically associated with a random hash function that takes the input (usually the data vector) and outputs a discrete value. Usually, this output serves as a (small memory) fingerprint which, being discrete, can be used for ‘smart’ indexing. These indices are most notably used for sub-linear time near-neighbor searches (31,32).

Sketching algorithms work by creating a dynamic probabilistic data structure popularly known as a *sketch* (33). The sketch is a small memory summary of a given set of items, which typically requires logarithmic memory for summarizing them (34). These sketches can support dynamic updates (35) and the dynamic query operation which returns an approximate estimate for a quantity of interest.

**Figure 2.**
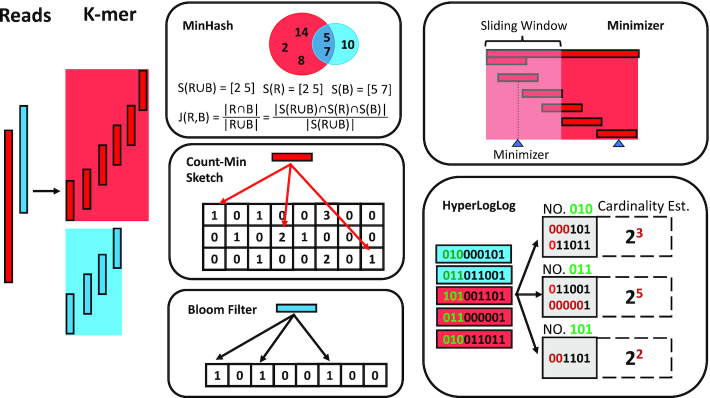
Important probabilistic algorithms and data structures. The sequences from two samples are decomposed into constituent *k*-mers. MinHash: each *k*-mer is hashed by a hash function and their hash values are stored in their corresponding hash sets *R* and *B*. MinHash sketch *S*(*R*) and *S*(*B*) with size *s* = 2 are shown. The Jaccard index between two hash sets is estimate by the fraction of hash values in the union of *S*(*R*) and *S*(*B*) (*S*(*R*∪*B*)) that are shared by both *S*(*R*) and *S*(*B*). Count-Min Sketch: three pairwise independent hash functions are applied to each *k*-mer. Each hash function is responsible for a row in the sketch and maps the hash values to the bins in its row. To encode an element into the sketch, the Count-Min sketch increases the numeric value in the mapped bins. To return the number of occurrences of a given *k*-mer, it hashes the *k*-mer using the same hash functions and returns the smallest value. Bloom filter: it initiates all the values in the array as 0. To record the presence of a *k*-mer in the dataset, it maps *k*-mer to the bits in the Bloom filter using three pairwise independent hash functions, and then it changes the mapped bits from 0 to 1. Minimizer: given a sequence, it can be compressed into a list of minimizers. To do that, a window slides across the sequence. In each window, the sequence inside the window is decomposed into *k*-mers. A minimizer is selected among the list of *k*-mers for the window at each position. HyperLogLog: each k-mer is represented by a hash value with length 9. The first three bits of a hash value is used to locate a register and the last 6 bits are saved in the corresponding register. The maximum number of leading zeros among all the values, that are stored in the register, is used to estimate the cardinality of each register.

To begin, we perform a concise overview of core probabilistic data structures and algorithms (Figure 2). We then include a review of a wide array of more recent variations, extensions, and recent advancements of these fundamental methodologies. Finally, we include a more in depth discussion on promising applications to genomic and metagenomic data.

### Fundamental algorithms and data structures

1. **Locality sensitive hashing** (LSH) was first introduced to solve the nearest neighbor search (NNS) problem in high dimensions (7). LSH functions are a subset of hash functions that seek to hash similar input values to the same hash values. Essentially, for an LSH function *f*, if two input items *x*_1_ and *x*_2_ are very similar to each other, then applying the LSH function to both should cause them to collide (*f*(*x*_1_) = *f*(*x*_2_)) with high probability. The main idea behind efficient retrieval is to use *f* to structure the data as an efficient dictionary or hash table by indexing data point *x*_*i*_ with key *f*(*x*_*i*_). Given any query *q*, *f*(*q*) naturally becomes a favorable key for lookup. This is because any *x*_*j*_ with the same key will have *f*(*q*) = *f*(*x*_*j*_), and hence, is likely to have high similarity with query *q*.
2. **MinHash** is arguably one of the most popular LSH functions for genomic and metagenomic data. MinHash takes a set as input and outputs a set of integer hash values. Specifically, MinHash applies *p* different hash functions to each element in a set and returns the minimal hash values from each of the *p* hash functions as the sketch of the set. The probability that two sets have the same minimal hash values is equal to the percentage of common elements in the union of both sets. As a consequence, we can quickly approximate the similarity between two sets by simply computing the ratio of the number of MinHash collisions between the sets and the total number of MinHashes. With MinHash we can compute a small approximate summary of each set, referred to as a sketch, and then calculate the similarity of any two sets as the distance between their sketches. Sequencing data are often conveniently represented as sets of tokens (or *k*-mers). As a result, MinHash is frequently used to quickly compare the similarity between two large sequencing datasets by applying the p hash functions to their *k*-mers.
3. **Minimizers** are another widely used technique within the family of LSH-algorithms to reduce the total number of *k*-mers for sequence comparison applications. A minimizer is a representative sequence of a group of adjacent *k*-mers in a string and can help memory efficiency by storing a single minimizer in lieu of a large number of highly similar *k*-mers. Minimizers will sample the sequence by choosing the smallest (lexicographically, for instance) *k*-mer within a sliding window. In Figure 2, the Minimizer portion demonstrates the sliding window that moves across the sequence, creating the set of minimizer *k*-mers for the sequence by taking the smallest *k*-mers within the window as it slides. The choice of the window length *w* and *k*-mer size *k* of the minimizers are parameters that can be adjusted for the application.

Several techniques employ hashing to compress the representation of a dataset. From these new representations, information can be rapidly queried.

1. **Bloom filter** (BF) is a data structure that compresses a set while still being able to query if an element exists in the set. The sketch for a BF is a bit array of *w* bits. The bits are given an initial value of 0. To record an element into the sketch, *p* different hash functions are used to map the input element to *p* different positions in the array. After evaluating the hash functions, the BF sets the bits to 1 at all mapped positions. To search for an element, the query element is hashed by the same *p* hash functions. Then, every bit that the hash values map to in the BF are checked. If any bit value of the mapped locations are not equal to 1, the input element is definitely not in the set. If all the mapped bits are 1, the element is likely in the set. This result can also be caused by random hash collisions while inserting other elements. Thus, the BF can have false positives. Ultimately, BFs can quickly evaluate the presence of a given element using very little memory.
2. **HyperLogLog** is designed to estimate the number of distinct elements in a set using minimal memory. The essence of HyperLogLog is to keep track of the count of the maximum number of leading zeros in the binary representation of each element in the set. If the maximum number of leading zeros observed is *n*, a crude estimate for the number of distinct elements in the set is 2^*n*^. This style of cardinality estimation only works for data distributed uniformly at random, so each element passes through a hash function before being evaluated and incorporated into an extremely compact sketch for the set. The process of cardinality estimation based on leading zeroes can have a high variance, so the HyperLogLog sketch distributes the hashed elements into multiple counters, whose harmonic mean yields a final cardinality estimation (after correcting for using multiple counters and hash collisions). But this memory is still logarithmic in the total number of distinct elements. On the other hand, calculating the exact cardinality requires an amount of memory proportional to the cardinality, which is impractical for very large data sets.

Alternatively, condensed representations may summarize the structure of the dataset by analyzing the frequency of components of the set. New datapoints that are assumed to exhibit the same structure can be efficiently acquired.

**Figure 3.**
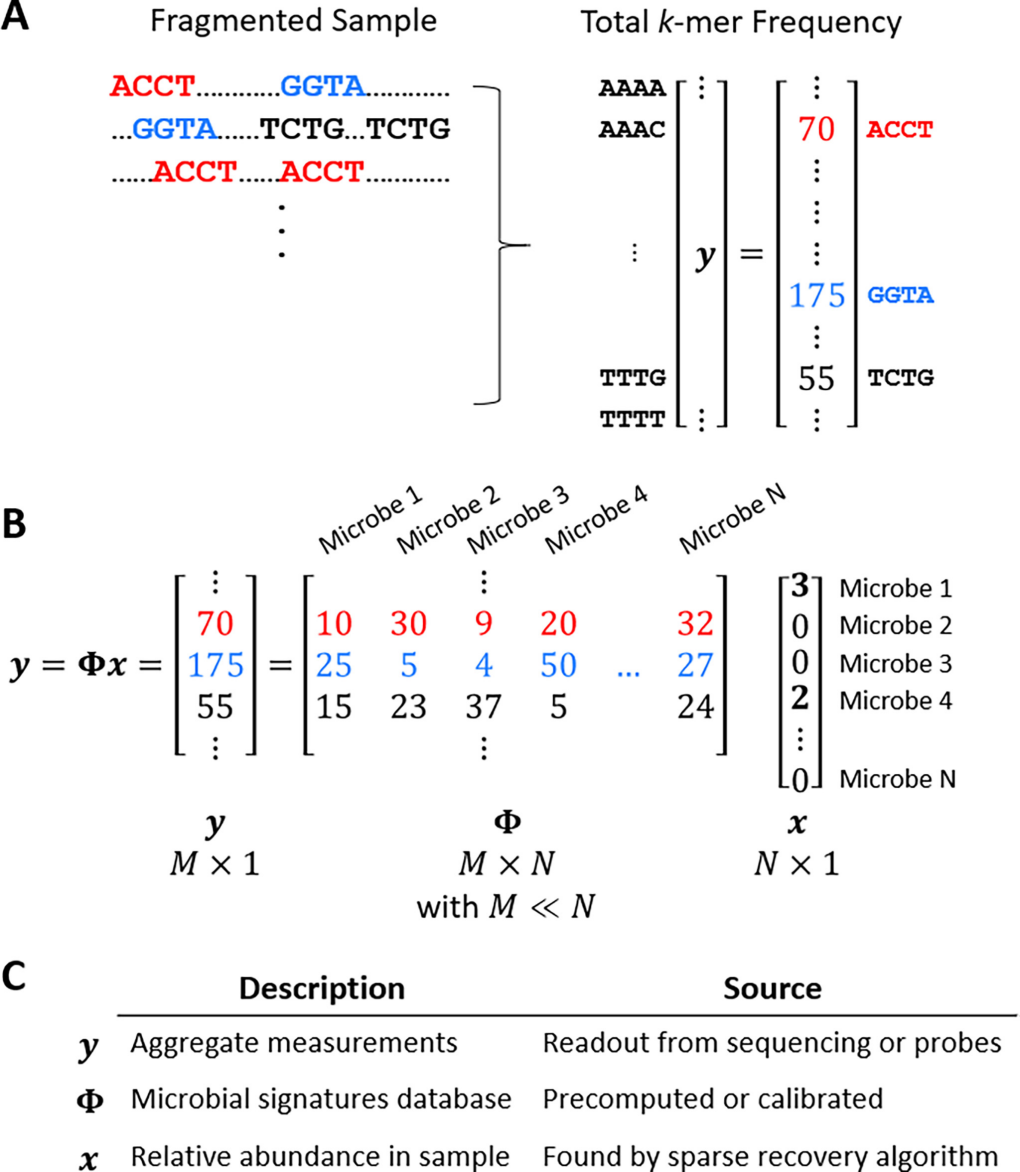
Example of CS for microbial quantification. (**A**) A nucleic acid sample is fragmented, and the *k*-mer frequency is calculated from sequencing data or inferred from probe binding events. *M* represents the number of *k*-mers chosen for analysis; in this simple example, all possible 4-mers are quantified for 4^4^ or 256 measurements. (**B**) Metagenomic samples contain nucleic acids from many microbes, so the measurement vector *y* represents a linear combination of underlying microbial quantities. Microbial signature *k*-mer frequencies are stored in *N* columns of a sensing matrix Φ in advance. Given Φ and having measured *y*, the goal of CS is to solve for *x*, the microbial quantities in the sample. To constrain the solution, CS assumes that the number of unique microbes in any given sample is far fewer than the *N* known microbes in the database, i.e. that *x* is sparse with many zero entries. Here, *y* is explained by a sparse combination of Microbes 1 and 4. (**C**) Summary of CS variables in the context of this application.

1. **Compressed sensing** is a signal processing technique that enables the acquisition of high-dimensional signals from low-dimensional measurements by leveraging the *sparsity* of many natural signals (16–18). Sparse signals have only a few nonzero elements. In metagenomics, a signal of interest may be the relative abundance of microbes in a sample. These signals are sparse because only a small fraction of all known species are present (i.e. have nonzero abundance) in any given sample. Figure 3 illustrates the process of CS in this context. The CS problem can be represented concisely with linear algebra: *y* = Φ*x* where an *M* × *Nsensing matrix* Φ captures an *N*-dimensional signal *x* with *M* linear measurements that are stored in *y*. Sparse recovery algorithms find the sparsest *x*′ that obeys *y* = Φ*x*′ either through a convex relaxation (e.g. a Lasso regression (16)) or a greedy algorithm (e.g., matching pursuit (36–39)). Theory shows that CS can make very efficient use of linear measurements; *M* scales *logarithmically* with *N* (17,18).
2. **Count-Min sketch** (CMS) is a specialized CS algorithm where the projection matrix Φ is a structured (0-1) random matrix derived from cheap universal hash functions. Due to this carefully designed matrix, it is possible to compute the projection *y* = Φ*x* as well as perform recovery of *x* from *y* without materializing the matrix in memory and instead only use a few universal hash functions, each of which needs only two integers. As a result, we get a provably logarithmic memory algorithm for compressing *x* and recovering its heavy elements. The CMS is popular for estimating the frequencies of different elements in a data set or stream.
  The CMS algorithm is remarkably simple and has a striking similarity with the Bloom filter. The CMS is a matrix with *w* columns and *d* rows. It can be thought of as a collection of *d* Bloom filters, one for each row, each using a single hash function. The only difference is that we use counters in CMS instead of bits in Bloom filters. Given an input data element *x* to the CMS, it is hashed by *d* independent hash functions. Each of the *d* hash functions generates a hash value hash_*d*_(*x*) within range *w* and increments the numeric value stored at column hash_*d*_(*x*) row *d*. Querying the count of an element consists of simply taking the minimum of the counters that the element hashes to in the CMS.

### Variations, extensions and recent advances

A tremendous amount of study and followup work has been performed by the scientific community to improve the fundamental probabilistic data structures and algorithms. Here, we give a brief overview of relevant variations, extensions, and recent advancements to the methodologies described above.

There has been a significant advancement in improving the computing cost of MinHash, which became a central tool in bioinformatics after the introduction of Mash (40) and other toolkits that then followed (41,42). Minhash requires *p* hash functions, and *p* passes over the data to compute *p* signatures. Recently, using a novel idea of *Densification* (43–45), Densified-Minhash was developed. Densified-Minhash only requires one hash function and one pass over the set to generate all the *p* signatures of the data with identical statistical properties as *p* independent Minhash, for any given *p*. Several improvements have been made for efficiently computing weighted Minhash as well (46), where the elements of sets are allowed to have importance weight. These recent advances have made it possible to convert data into Minhashes in the same cost as data reading, which, otherwise, was the main bottleneck step.

Genomic applications also use many LSH functions beyond MinHash. Simhash (47) was invented by Google to find near-duplicates over large string inputs using cosine similarity. It was shown in (48) that for sequence and string datasets Minhash is provably and empirically superior to Simhash, even for cosine similarity. B-bit minwise hashing is a variation of MinHash that saves only the lowest b bits of each hashed value (49). It requires less memory to store each hash code and can be used to accurately estimate the similarities among high-dimensional binary data. Sectional MinHash (S-MinHash) (50) includes information about the location of k-mers or tokens in a string to improve duplicate detection performance.

Universal (or random) hash functions seek to quickly and uniformly map inputs to hash codes. Universal hash functions are important building blocks for the CMS, Bloom filter, hash table, and other fundamental data structures. MurmurHash (https://sites.google.com/site/murmurhash, Accessed March 2020) is a very well-known universal hash that has been widely used in many bioinformatic software packages, including Mash (40). Although previous MurmurHash versions were vulnerable to hash collision, Murmurhash3 (https://github.com/aappleby/smhasher/wiki/MurmurHash3, Accessed March 2020) is a good general-purpose function that is particularly well-suited to large binary inputs. However, there are other options such as xxHash (https://github.com/Cyan4973/xxHash, Accessed March 2020), which can be faster than MurmurHash, and CityHash (https://opensource.googleblog.com/2011/04/introducing-cityhash.html, Accessed March 2020). CityHash is relevant to genomics because it is optimized for strings. It outperforms MurmurHash for short string inputs but is appropriate for any length input. FarmHash is the successor to CityHash and also focuses on improved string hashing performance (https://opensource.googleblog.com/2014/03/introducing-farmhash.html, Accessed March 2020). ntHash (51) is a specialized DNA hashing function. It recursively calculates the hash values for the consecutive k-mers in a given sequence. While ntHash can be faster than xxHash, CityHash and MurmurHash, it is only appropriate for sequence data.

Minimal perfect hash functions (MPHF) and perfect hash functions (PHF) map inputs to a set of hash codes without any collisions. A PHF maps *N* inputs, or keys, to a set of >*N* hash codes, some of which are unused. An MPHF maps *N* inputs to *N* codes. Although MPHFs have been used to improve many bioinformatics applications, such as the quasi-dictionary (52), the MPHF construction process is often resource-intensive. Critically, all of the inputs must be known in advance to construct an MPHF, and many construction methods based on hypergraph peeling fail to scale. BBhash is an MPHF construction method that was introduced to scale to massive key sets (53). BBhash is constructed by a simple procedure that maps each key to a fixed-size bit array using a universal hash. If two keys collide in the bit array, the corresponding location is set to 1. Otherwise, the bit remains 0. This recursive process is repeated with all of the colliding keys until there are no more collisions. Due to the simplicity of the algorithm, BBhash construction is much faster at the scale typically encountered in genomics.

MPHFs are usually used to implement fast, read-only hash tables with constant-time lookups. However, clever open addressing schemes can also be used to achieve similar query performance without knowing the key set in advance. Rather than avoid hash collisions, open addressing attempts to rearrange elements in the hash table for optimal performance. For instance, hopscotch hashing (54) ensures that a key pair is always found within a small neighborhood of its hash code. Since only a small collection of consecutive buckets need to be searched when a query is issued, hopscotch hashing has very strong query-time performance. Robin Hood hashing (55) is another open addressing method. The key feature of this algorithm is that it minimizes the distance between the hash code location and the actual key-value pair, reducing worst-case query time. Cuckoo hashing (56) uses two hash functions and guarantees that the element will always be found at one of the two hash indices.

Some fundamental advances in LSH have also been seen with minimizers. Traditionally, minimizer selection is executed according to lexicographic order. However, this procedure may cause ‘over-selection’ where more *k*-mers than necessary become minimizers. Instead, researchers recently proposed to select minimizers from a set of k-mers based on a universal hitting set or a randomized ordering (57). If minimizers are picked from the universal hitting sets, which are the minimum sets of k-mers that cover every possible L-long sequence (58), the expected number of minimizers in a given sequence would decrease.

There is also recent progress in techniques to rapidly characterize datasets. HyperLogLog has risen to prominence recently thanks to its ability to efficiently count distinct elements in large data sets and databases. Many new algorithms have since been developed based on HyperLoglog to adapt to different scenarios. For instance, HyperLogLog++ (59) was introduced to reduce the memory usage and increase the estimation accuracy for an important cardinality range. Sliding HyperLogLog (60) adds a sliding window to the original algorithm for more flexible queries, but it requires more memory storage.

Bloom filters are attractive because they can substantially compress a dataset, but this approach can return false positive answers. Cascading Bloom filters (61,62) improve the accuracy of the standard Bloom filter. A cascading Bloom filter recursively creates child Bloom filters to store the false positives from a parent Bloom filter. This reduces the false positive rate (FPR) of the overall system at a small memory cost. An alternative FPR reduction strategy is the *k*-mer Bloom filter (kBF) (63). Each *k*-mer in a sequence overlaps with its adjacent *k*-mers by *k* − 1 base pairs. Therefore, the existence of two *k*-mers in a sequence is not independent, and the presence of a particular *k*-mer in the Bloom filter can be verified by the co-occurrences of its neighbors. Based on this information, *k*BF lowers the FPR by checking, for instance, the query’s eight possible neighboring *k*-mers (four to the left and four to the right). If none of the query’s neighbors exist in the Bloom filter, *k*BF rejects the query as a false positive.

There are also many algorithms built around the generalized Bloom filter data structure. These methods give the Bloom filter different functions, but maintain its simplicity and memory-efficiency. The counting bloom filter (CBF), for instance, was developed to detect whether the count of an element is below a certain threshold (64). The only difference between the BF and CBF is that when adding an element, all the counters for that element increase by 1. The Spectral Bloom filter (SBF) (65) functions similarly to a CBF, but the SBF only increases the minimum value in the table when inserting an element. This modification causes SBF to have a lower error rate when compared to the CBF.

In addition to extensions and variations of fundamental methods, recent advances have developed by combining several core data structures and techniques. For instance, RACE (66) is an algorithm to downsample sets of genetic sequences while preserving metagenomic diversity. RACE replaces the universal hash function in the CMS with an LSH function. Using MinHash, RACE can identify frequent *clusters* of sequences rather than frequent elements. Since RACE is robust to sequence perturbations, it can be used to implement diversity sampling. By adjusting the LSH collision properties, RACE can create a sampled set of sequences that retains metagenomic diversity while substantially downsampling a data stream.

The RACE diversity sampling algorithm is attractive because it can downsample accurately with high throughput, low memory overhead, and only one online pass through the dataset. For each sequence in an input stream, RACE checks to see whether the sequence belongs to a frequent cluster. This is done by replacing the minimum operation in the CMS with an average over the count values. Due to a deep connection between RACE and kernel density estimation, the average is a measure of the number of nearby sequences in the dataset, otherwise known as a density estimate. If the density is low, then RACE has not seen many similar sequences and the sequence is kept. Otherwise, the sequence is discarded. In theory and practice, RACE attempts to select a constant number of sequences from each cluster. When MinHash is properly tuned to differentiate between species, the clusters in the RACE algorithm correspond to different species in the dataset. As a result, RACE provides a fast, online and robust way to downsample sequence datasets while retaining important metagenomic properties.

Another important development comes from the CMS and Bloom filters. RAMBO (Repeated and Merged Bloom Filter) (67) is a recent development in multiple set compression for fast *k*-mer and genetic sequence search. The RAMBO data structure is inspired by the CMS, but the goal is to report the sequence containment status rather than sequence frequency. RAMBO consists of a set of *B* × *R* Bloom filters. Rather than maintain one Bloom filter for each set of k-mers, RAMBO uses a 2-universal hash function to randomly merge *K* datasets into *B* groups ( 2 ≤ *B* ≪ *K* ) so that each group has approximately *K*/*B* datasets. Each partition is compressed using a Bloom filter. This process is independently repeated *R* times with different partitions. To determine which sets contain a query sequence, RAMBO queries each Bloom filter. Because the groupings are random, each repetition reduces the number of candidates by the factor 1/*B* until only the correct datasets are reported at the end of the algorithm. The key insight is that *B* × *R* ≪ *K*.

With this approach, RAMBO can determine which datasets contain a given k-mer or sequence using far fewer Bloom filter queries, yielding a very fast sublinear-time sequence search algorithm (68). RAMBO also inherits many desirable features from the CMS and the Bloom filter. This includes a low false positive rate, zero false negative rate, cheap update process for streaming inputs, fast query time, and a simple systems-friendly data structure that is straightforward to parallelize.

In addition to methods that enable the scalable *processing* of high dimensional data, there are fundamental extensions of and considerations for CS that enable its efficient *acquisition*. While applications of CS are constrained to those where the sparsity assumption is appropriate, seemingly irrelevant signals may have a hidden sparse representation in some *basis*. For example, JPEG image compression exploits the fact that natural images can be sparsely represented (or at least approximated) in a discrete cosine basis (a cousin of the Fourier transform). When the sparsity basis is known in advance, the canonical CS problem can be reformulated from *y* = Φ*x* to *y* = ΦΨ*s* where *s* is the sparse representation of *x* in the basis defined by the columns of Ψ. This transformation was recently demonstrated in transcriptomics (69) and may soon find an analogous application in metagenomics.

Aside from signal sparsity, CS also imposes constraints on the sensing matrix. Specifically, Φ must adequately preserve signals’ separation distances; highly distinct *N*-dimensional signals should not be forced into close proximity in *M*-dimensional space once projected by Φ (70,71). While Gaussian and other classes of random matrices have been shown to work well in the general case, recent techniques indicate that Φ can be iteratively optimized for a given task by simulating measurements and sparse recovery of signals (72). However, as we discuss below, practitioners generally do not have full control of Φ in most applications. In metagenomics, the values in Φ are constrained by the nucleic acid content of natural organisms. Because each chosen sensor makes up a row of Φ, a new algorithm can select *M* sensors (e.g. *k*-mers or probes) from a set of options to optimize the properties of Φ for CS (73).

Very recent techniques in CS are also exploring how to merge machine learning with CS. Given a dataset, recent work indicates that both the sensing matrix Φ and the procedure that recovers *x* from *y* = Φ*x* can be learned from specially designed deep neural networks (74–77), even in cases where the signal’s sparsity structure is nonlinear. Datasets in metagenomics are known to be highly structured and could thus be positively impacted by these recent advances in CS in the near future.

## APPLICATIONS TO METAGENOMICS

Most, if not all, of the approaches described above have found their way into previously published bioinformatics methods. However, method development to date has been primarily focused on genome sequencing for a single individual or isolate genome. Findings suggesting links between microbiomes, such as the human gut microbiome, and human disease (78,79) has led to increased metagenomic sequencing. The rapid growth of this type of sequencing, where the set of reads is from a complex community of organisms, adds additional complexity and new challenges to fundamental comparative genomics problems. Here we list a core set of these fundamental problems faced when performing metagenomic sequence analysis: (i) sequence resemblance, (ii) sequence containment, (iii) sequence classification, (iv) sequence downsampling, (v) sequence profiling, (vi) sequence probe design. For each problem, we discuss the role of the previously described approaches and newer tools incorporating recent advances (Table 2).

**Table 2.** Metagenomics software based on probabilistic and signal processing algorithms. Six main application areas are highlighted: containment, downsampling, probe design, profiling, resemblance and taxonomic classification. Speed indicates the relative computational speed of CPU operations, memory the relative maximum RAM used during index construction/query steps and year the publication year. More ‘⋆’s means better time and memory efficiency. Less ‘⋆’s indicate more resource intensive tools. Performance estimates using only literature based comparison are marked in gray (‘⋆’). The stars (1-5) correspond roughly to time (days, hours, minutes, seconds and milliseconds) and memory (>64GB (server), >16GB (workstation), >1GB, >16MB and <16MB). Datasets used were Shakya *et al.* (133) (Downsampling, Profiling and Taxonomic Classification), 99 sequencing experiments from SRA (132) (Containment), 1028 *E. coli* genomes from NCBI Refseq (134) (Resemblance) and a dataset containing Coronavirus, West Nile virus, Zika virus, Yellow Fever virus and Ebola virus genomes from NCBI RefSeq (134) (Probe Design). Tools supporting multithreading were run with 30 threads. KrakenUniq and Kraken2 were run on their standard databases and are expected to show better memory efficiency if MiniKraken DB is chosen instead. BioBloom Tools and Opal were indexed using the training data provided by Opal which is much smaller than the DBs other tools use. MetaMaps is a classifier specifically for Long Read sequences as compared to the other tools in the category. The datasets and results for each tool can be found at https://gitlab.com/treangenlab/hashreview

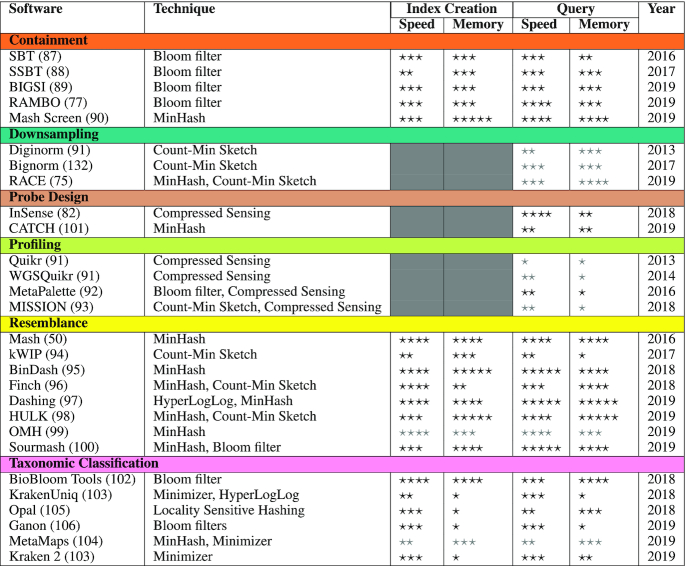

### Sequence and metagenome resemblance

One of the recent breakthroughs in the area of large-scale biological sequence comparison is in the use of locality-sensitive hashing, or specifically MinHash and Minimizers, for efficient average nucleotide identity estimation, clustering, genome assembly, and metagenomic similarity analyses.

**Mash**. In response to the high computational expense of large-scale sequence similarity calculations, researchers have begun to apply probabilistic approaches such as using MinHash to approximate the similarity between sequences (6). In the seminal work of Mash (40), it was shown that MinHash could be used as an extremely efficient estimator for genome similarities in both speed and resource use. It was also shown how Mash could be applied to similarity estimates between entire metagenomes. In addition, Mashtree has experimented with building phylogenetic trees based on the genomic similarity estimated using Mash (80). These and other applications led to a quick and widespread adoption of Mash throughout the research community for rapid sequence similarity calculations.

Despite representing a paradigm shift, one of the shortcomings of MinHash is that its similarity estimation is most accurate when the two sets have similar sizes and their intersection region is large (81). In the paper (82), the authors also point out that the genomic similarity estimated via Jaccard distance is sensitive to the data set size. Another limitation of MinHash applied to metagenomics is that large amounts of rare *k*-mers can dominate the sample sketches. These *k*-mers which only occur a few times could be the result of sequencing errors as well as being actual rare species present in a metagenome. We will now review several other recent bioinformatic tools that have accelerated sequence similarity in the era of terabyte-scale datasets.

**BinDash** (83), like Mash, takes in sequences, compresses them into sketches and then compares sketches to estimate the genome similarities. Specifically, BinDash focuses on accelerating the sketch construction and sketch comparison time. To do this, BinDash uses the b-bit one-permutation MinHash algorithm to compress sequences. Given a sequence, BinDash first decomposes the sequence into *k*-mers. Each *k*-mer of the sequence is hashed by one predefined hash function. The hash values of *k*-mers are then pooled together into *B* buckets. After all the *k*-mers are hashed and then grouped into *B* buckets, BinDash selects the smallest hash value from each bucket and stores the *b* lowest bits of each selected hash value as the sketch of a sequence. To account for potentially empty buckets, the sketch process is optimized by the densification operation as mentioned in the previous section. The sketch similarities are then estimated using Jaccard indices based on the *B* · *b* bit sketch. The experiments show that, comparing to Mash, BinDash can characterize the same data set with less error, less memory used and faster speed.

**Dashing**. The recently introduced work of Dashing uses HyperLogLog (HLL) sketching to approximate genomic distances (84). One main motivation behind Dashing is to improve the similarity estimation accuracy across input sequence datasets with different sizes. Dashing represents the first time that HLL has been applied to estimate the overall similarity between sequence samples. Given that HLL is used to estimate set cardinality, to use HLL to estimate genomic sequence similarities you must estimate the intersection of the two sequence data sets’ *k*-mers, then estimate the cardinality of this intersection set. Dashing first sketches the *k*-mers of each given sequence data set using HLL. It then creates a union sketch using basic register maximum operations between the two HLL sketches. Now, having access to the set cardinality of both independent sets, as well as the union set size, the inclusion-exclusion principle yields the set cardinality of the intersection between the two sequence datasets. The HLL set cardinality calculations of Dashing are estimated using a maximum-likelihood-based approach, which has higher accuracy than the traditional corrected harmonic mean estimation approach. Dashing is able to sketch metagenomes faster than previous approaches, but it requires more CPU time to calculate the genomic distances. In the end, comparing to Mash, Dashing has faster speed, higher accuracy and a lower memory footprint. **Finch** Rare *k*-mers can distort the estimation of sequence comparisons and inter-metagenomic distances. To solve this problem, Finch (85) uses MinHash with a larger sketch size in order to evaluate the abundance of each *k*-mer. It then decides thresholds based on estimated abundances to filter out low abundance *k*-mers. It also removes *k*-mers with unequal frequencies of forward and reverse sequences. By deleting erroneous or rare *k*-mers, Finch can estimate the distances between metagenomic samples robustly. Finch also reports including correction for sequencing depth biases.

**HULK** estimates the similarities among metagenomic samples while taking *k*-mer frequencies into account (86). In HULK, a metagenomic sample is sketched via histogram sketching (87) into a final histosketch, which preserves *k*-mer frequency information. To build a histoskech for a given metagenome, reads are first decomposed into *k*-mers and then streamed in a distributed fashion into independent Count-Min sketch counters. Once a large number of reads have been counted, HULK sends the CMS data to be histosketched and resets the CMS counts to initial values. In order to create the final histosketch, HULK first summarizes the Count-Min sketch counters into a *k*-mer spectrum and then applies consistent weighted sampling (https://www.microsoft.com/en-us/research/publication/consistent-weighted-sampling/, Accessed March 2020) methods. HULK can successfully cluster metagenome samples based on similarity between histosketches as well as being a faster approach than that of naive *k*-mer counting.

**kWIP** is yet another recent approach that tries to improve the accuracy of estimating sequence dataset similarity via *k*-mer weighted inner product (kWIP) (88). kWIP first uses khmer (89), which is a *k*-mer counting software relying on Count-Min Sketch, to compress each metagenomic read sample into a sketch. Each sketch is an array consisting of *m* bins. Each bin is responsible for counting the number of occurrences of some of the *k*-mers (with collisions) in the sample. To calculate the distance between two samples, each of the *m* bins is assigned a weight to be used in a weighted inner product. In order to assign weights to individual bins, kWIP first counts the number of non zero bins across all of the *n* samples. An *m* length vector containing these frequencies is then used by kWIP to create another *m* length vector converting the frequency values to a new value based on Shannon entropy. This entropy conversion causes bins that have k-mers present in roughly half of the samples to be heavily weighted versus bins that have k-mers present in all or none of the samples (which get a weight of zero). Genetic similarity is then approximated by the kWIP distance. The kWIP distance is calculated using the inner product between two sample sketches, with each bin weighted by the Shannon Entropy for that bin. The authors show that kWIP can produce more accurate results than Mash, especially for metagenomic samples with low divergence. Of note, kWIP is specifically designed to create a distance matrix from multiple samples, using all samples in the sketching process, as opposed to comparing individual sketches for individual samples like most other methods discussed here.

**Order Min Hash (OMH)** introduces a new way of sketching a sequence that estimates the edit distance of the sequences. (90) Unlike most other hashing based techniques for similarity calculations, which treat all the k-mers without respect to the order in which they occur, OMH preserves the *k*-mer ordering in its sketching process. The sketch for a given sequence consists of *n* vectors of length *l*. Each of the *n* vectors contains *l* representative *k*-mers, which are selected according to a pre-defined permutation function, and whose relative ordering is maintained from the original sequence. The distance calculation uses the weighted Jaccard distance, where the number of appearances of a *k*-mer are taken into account.

**Sourmash** (42) is closely related to Mash and based on MinHash. It modifies the sketching procedure such that the sketch size can be of variable length for different sequences. In their approach, the size of the sketch is based around the number of unique *k*-mers unlike the fixed size MinHash sketch. Additionally, sourmash includes functionalities such as *k*-mer frequency calculations as well as a sequence containment method that combines the Sequence Bloom Tree and MinHash methodologies.

### Metagenome containment

Searching for the containment of a read, gene fragment, gene, operon, or genome within a metagenomic sample or sequence database is a frequent computational task in bioinformatics. This is an open challenge for two key reasons: first, the size of metagenomic and sequence repositories are on the scale of terabytes to petabyes. Thus, methods able to quickly eliminate all the non-matching sequences in the database are crucial. Second, sequences evolve over time and rarely, if ever, will be an exact match especially as metagenomes and sequence databases contain a huge amount of sequence diversity. Methods that tolerate mismatches and indels have much improved sensitivity compared to methods that require more strict exactly matching sequences to satisfy containment. Despite the breakthroughs made via Bloom Tree inspired structures in sequence search, these approaches are not without drawbacks. First, they have to make a trade-off between false-positives and the filter size due to the inherent limitations of the Bloom filter. Second, they commonly lack flexibility; once the filter size is determined, they cannot be changed based on the size of the input sequences. No matter how many *k*-mers a sequence has, they all have to be sketched into a fixed size array. Finally, as the size of the input data increases, the precision of the Bloom filter-based sequence search typically declines. We will now review a few recent approaches that have tackled this important task in computational biology.

**Sequence Bloom Tree (SBT)** (91) is a binary tree where each node in the tree is a Bloom filter. An SBT is used to index large sequence databases for efficient containment check of a query sequence within the database sequences or datasets. To construct an SBT, each sequence or dataset is added one by one, beginning with adding the first dataset as the root of the SBT. For each additional sequence or dataset, you first compute the Bloom filter for the contained *k*-mers, and then scan from the root of the SBT to the leaves, inserting the dataset’s representative Bloom filter at the bottom of the tree. At each bifurcation, the insertion traversal follows the path of the child with the closest Hamming distance similarity to the Bloom filter for the current dataset. After insertion is finished, the new dataset’s Bloom filter is added as a leaf node, and each node in the SBT contains the union of the Bloom filters of its children. To be specific, if a *k*-mer is present in node *u*, it should also exist in all the direct ascending nodes’ Bloom filters from *u* to the root. Therefore, as a Bloom filter gets closer to the root, it becomes more populated and the false-positive rate of the Bloom filter is higher (a process known as saturation). Querying for sequence containment proceeds by querying each node’s Bloom filter, starting with the root, and determining if enough *k*-mers are contained from the query’s *k*-mers. If the Bloom filter contains enough of the query’s *k*-mers, then each child node’s Bloom filter is queried for containment. The process proceeds until each sequence or dataset containing the query at the leaves of the SBT is determined.

**Split sequence bloom tree (SSBT)** (92) were implemented to quickly search short transcripts within a large database. Although the SSBT was originally designed for RNA-seq data, it can be adapted to other sequence containment problems just like SBTs. The SSBT is an improvement over the sequence Bloom tree (SBT) data structure (91). Similar to SBTs, each sequence or dataset in the database is inserted into the SSBT by traversing from the root of the tree to the bottom. The SSBT is also a binary tree, but each node has two Bloom filters instead of one. The first filter, called the *similarity filter*, saves *k*-mers shared by all the datasets in the subtree under a particular node. The second filter, named the *remainder filter*, stores the k-mers that are not universally shared among all the datasets but are specific to at least one dataset in the subtree for a node. The union of the *similarity filter* and the *remainder filter* is a single Bloom filter for the node similar to the nodes of an SBT. SSBT is a clever re-organization of SBT resulting in accuracy similar to an SBT but with reduced space occupancy and search time.

**BIGSI** represents a significant advance in sequence containment search; BIGSI was introduced to allow efficient search for a query sequence among a large bacterial and viral genome database (93). It also relies on Bloom filters to solve this problem. But, instead of using a tree-like structure (e.g. SBT), BIGSI employs a flat Bloom filter-based data structure. BIGSI first indexes the reference datasets, where these datasets are raw FASTQ read datasets or assemblies from which to search for the presence of a query sequence. To index the reference datasets, BIGSI first extracts a set of non-redundant *k*-mers from each dataset, and then builds a corresponding Bloom filter. After this initial step, BIGSI then concatenates all the Bloom filters together. BIGSI compresses the whole database into a matrix, in which each column is a Bloom filter for a given dataset. To conduct an exact search of a sequence, BIGSI is expected to find the index of all the *k*-mers of the query sequence inside the matrix. For inexact search, as referenced above, BIGSI just needs to find the index for a subset of the *k*-mers present in a sequence of interest. BIGSI can also dynamically update the size of the sketch based on the amount of input datasets. When new datasets arrive, BIGSI can add a new column to the matrix for each new dataset.

**RAMBO** (68) is a very recent method which also allows indexing new sequences and new datasets in a streaming fashion. Contrary to BIGSI, which has *O*(*K*) (*K* is the number of datasets) query time, RAMBO is sublinear in query time with a slight increase in memory.

**Mash Screen** (94) was developed to determine which reference sequences are contained within a metagenomic sample using MinHash, though the methodology is also presented as a method for sequence similarity. Similar to MetaPallette (described below), it uses references found to be contained in a metagenome to describe the metagenome’s taxonomic composition, but does not classify individual reads. Mash Screen first converts a reference sequence and a given metagenomic sample into two sets of *k*-mers *A* and *B*. Following that, Mash Screen compresses the set of reference *k*-mers *A* into sketch *S*(*A*) using MinHash. It then creates π(*B*) by hashing every *k*-mer in *B* and checks if each hashed *k*-mer intersects (is contained) in *S*(*A*). The calculation of $\frac{| S(A) \cap \pi (B) |}{| S(A) |}$ represents the fraction of k-mers in the sketch of *A* contained in *B*, and is referred to as the containment index. Finally, the containment index is converted to a score that approximates sequence similarity. This final score is referred to as the Mash containment score. The presence or absence of one or more reference sequences in a metagenomic sample is then determined by this Mash containment score. An example is given, for instance, of searching for a set of reference viral sequences in hundreds of metagenomes by calculating the Mash containment score between each reference and each metagenome.

### Metagenome classification

Metagenomic sequence classification software typically uses reads to search against known genomes and perform lowest common ancestor based taxonomic classification. As the size of the reference databases (terabytes to petabytes) and the number of reads (10s of millions to billions) in metagenomic samples increase, it becomes computationally intractable to perform exhaustive comparison of all *k*-mers in the reads against all *k*-mers within the reference databases, opening the door for efficient new tools. Tools like Kraken (95) and DIAMOND (96) were two of the first ultra efficient tools for fast metagenomic classifications. We now review a few recently developed approaches for metagenomic sequence classification.

**KrakenUniq** is built based on Kraken and its main goal is to decrease the false-positive read classification rate (97). Compared to Kraken, one of the additional features of KrakenUniq is that the number of unique *k*-mers of each taxon is recorded while processing all reads of a metagenomic data set. KrakenUniq uses HyperLogLog to efficiently estimate these unique *k*-mer counts. By tracking the number of unique *k*-mers for a taxa alongside the coverage for that taxa across all the reads in a metagenome, KrakenUniq can identify likely false-positive read classifications caused by events such as sample contamination, low-complexity regions, and contaminated database sequences.

**Kraken2** substantially reduces memory usage, while simultaneously gaining a significant boost in classification speed, when compared with Kraken 1 (98). This advancement in memory use and speed comes from using a compacted hash table that stores LCA assignments for hashed minimizers of *k*-mers instead of a table storing LCA assignments for all k-mers as in Kraken 1. While this hash table saves significant memory, it comes at a small specificity and accuracy cost given that it only stores pairs of minimizers and LCAs which are further subsampled through hashing. This hashing process includes adding spaced seed masking to the minimizer before hashing. The size of this new compact hash table can be specified by the user, with smaller sizes reducing the memory footprint and increasing speed but lowering classification accuracy. When compared with other state of the art tools, Kraken2 ultimately provides similar or better classification accuracy alongside its memory and speed improvements.

**BioBloom Tools (BBT)** (99) is novel in that it applies a multi-index Bloom Filter (miBF) to the sequence classification problem. The miBF is a Bloom filter-like data structure that consists of three arrays. The first array serves as a traditional Bloom filter, recording the existence of hashed items in a set. The second array, named the rank array, tracks the number of non zero bits stored in the first Bloom filter array at certain intervals (by default, the number of non zeros every 512 bits in the Bloom filter is stored). To reduce memory usage, the rank array is ultimately interleaved with the first Bloom filter. The third array, also referred as the ID array, saves the integer identifiers (IDs) for reference sequences inserted into the miBF. These IDs allow the miBF to additionally store associated taxonomic classification information for entries so as to be used as a classifier.

For each reference sequence, BBT hashes spaced seeds into the miBF rather than contiguous *k*-mers. Spaced seeds, unlike *k*-mers, allow mismatches between the references and the queries which can increase the sensitivity of approximate sequence search (100). To classify a given read, spaced seeds from the read are looked up in the Bloom filter. The rank array is then used to help retrieve IDs from the ID array. Ultimately, the retrieved IDs lead to a final taxonomic classification. To reduce the false positive rate, BBT makes use of nearby spaced seeds within adjacent sliding windows, referred to as frames, when performing its classifications. BBT also intelligently populates the ID array in multiple passes such that the effects of data loss from hash collisions is minimized.

**Ganon** (101) focuses on quick database indexing in order to ensure usage of the most up to date sequence database data to accurately classify reads. Many existing tools apply static, out-of-date versions of databases to assign reads. This approach can miss, for instance, classifications for species that have been newly sequenced and very recently added to existing databases. To overcome this problem, Ganon employs Interleaved Bloom Filters (IBF) (102) to index up-to-date reference genomes efficiently. An IBF is an array of length *b* · *n*. It encompasses *b* Bloom filters of length *n*. To index the references, Ganon first groups the sequences into clusters. These clusters should roughly mirror different groups for a given taxonomic rank such as different species or strains. It then sketches each cluster into a single Bloom filter. Lastly, all the Bloom filters are interleaved into one IBF. Reads are classified that pass a minimum threshold for the number of matches found within the read and the references. If a given read can map to multiple references, an optional lowest common ancestor (LCA) approach can be applied.

**MetaMaps** was designed to perform classification on noisy long read data including making both classifications and abundance estimates down to the strain level (103). MetaMaps classifies long reads by mapping them to reference genomes. Given that reads could map to many closely related references, metamaps simultaneously performs mapping as well as estimating the community composition of a metagenome sample. Thus, when determining the probability of mapping a read to a reference, the probability is a combination of both a probabilistic mapping quality to the reference as well as the estimated abundance of the reference’s taxonomic unit in the sample. To quickly find mapping locations for reads across all reference genomes, an efficient probabilistic approach is used that generates initial candidate mappings using minimizers followed by a winnowed-minhash statistical modelling approach for further ANI estimation (104). The read mappings and metagenome abundance estimates are then iteratively updated through an Expectation-Maximization (EM) algorithm.

**MetaOthello** (105) is one of the latest efforts in improving the classification speed of metagenomic classification. Similar to Kraken2, MetaOthello reports significant improvements in both memory use and speed when compared to, for instance, Kraken 1. MetaOthello applies the recently developed l-Othello data structure to speed up the process, which is a hashing based classifier. MetaOthello uses *k*-mers that act as signatures for taxa to make its classifications. A *k*-mer is a signature for a taxon if it is only present in that taxon or that taxon’s subtree, and nowhere else in the tree of life (it is taxon specific). MetaOthello indexes all reference sequences, finds all taxon signature *k*-mers and their taxonomic mappings, and populates an l-Othello data structure that efficiently maps from signature k-mers to taxa. The l-Othello, once built, maintains two arrays *A* and *B* populated with binary values. When looking up a *k*-mer’s taxa mapping in the l-Othello, the k-mer is hashed by two hash functions *h*_*a*_ and *h*_*b*_ that map to the matching positions in *A* and *B*. The final corresponding taxa value *t* for the *k*-mer is calculated through a bit-wise XOR operation of the two values found in *A* and *B*. Thus *t* = *A*[*h*_*a*_(*s*)]⊕*B*[*h*_*b*_(*s*)].

The classification step of MetaOthello operates similarly to other approaches. A query sequence is decomposed into its constituent *k*-mers and the corresponding taxa for each *k*-mer is looked up using the l-Othello data structure. Then, differing from other approaches, MetaOthello uses a windowed approach to make the final classification. For a given taxonomic rank, the classification takes into account the maximum number of contiguous taxa assignments that all occur consecutively within the query sequence.

**Opal** (106) is an LSH-based metagenomic classifier that uses Low Density Parity Check (LDPC) codes. The rationale for using an LDPC LSH approach is to ensure even coverage for all of the positions in the *k*-mer while using as few hash functions as possible. The authors highlight that this is the first application of low-density LSH in bioinformatics. The rationale for using low-density LSH is that it will avoid coverage bias issues and offer increased accuracy when using long *k*-mers.

### Downsampling

In addition to newer more efficient methods for analyzing large metagenomic data sets, a parallel effort has been emerging that instead reduces the data set size first before running further downstream analyses. Intelligently down sampling, for instance, a read data set can dramatically speed up any further computations performed, while ideally preserving the important characteristics of the metagenome. Another alternative approach to analyze less data than a full metagenome would be to restrict sequencing to a small subset of regions in the metagenome such as the 16S rRNA. This sequencing approach, referred to as metabarcoding (107) or amplicon sequencing, can help to simplify other downstream tasks such as community profiling and taxonomic assignments of reads. Here, however, we consider only the recent computational approaches that shrink large metagenomic datasets previously generated or in an online streaming fashion.

**Diginorm** (108) is a CMS-based method for downsampling shotgun sequencing data. Diginorm is a streaming algorithm that can select a small set of reads from a large dataset using relatively few computational resources without substantial information loss. This improves the speed of downstream tasks. Diginorm begins by finding the frequencies of all *k*-mers in a sequence using a CMS. If the median frequency value is larger than a threshold, usually 20, the sequence is discarded. This process discards reads with *k*-mers that have already been observed in other reads. Since rare reads have many rare *k*-mers, they will have a lower median count than common reads and will be kept. An easy-to-use Python implementation is provided in the khmer package.

**Bignorm** (109) is an extension of the ideas behind Diginorm. Bignorm obtains better downsampling performance by including additional information, such as quality scores and common error modalities, when determining whether to accept a read. While Bignorm is still based on *k*-mer abundance counts and the CMS, the decision threshold is based on a weighted summary of *k*-mer counts rather than simply the median. The decision process attempts to remove bias in Diginorm that may incorrectly accept a read. For instance, Bignorm attempts to differentiate between rare *k*-mers caused by single substitution errors and authentic uncommon reads. While Diginorm and Bignorm are both efficient streaming algorithms, Bignorm is implemented in C++ and uses parallelism to achieve faster processing times.

**RACE** (66) is a recent downsampling method based on LSH and the CMS. Rather than consider explicit *k*-mer abundance statistics, RACE is based on Jaccard similarity. Diginorm and Bignorm both discard reads which contain many *k*-mers that have already been observed. RACE discards reads that have a high Jaccard similarity with many observed reads. While these decision criteria are similar, density estimation with Jaccard similarity is incredibly efficient using the RACE algorithm.

### Metagenome profiling

**Quikr/WGSQuikr** (110,111) are CS-based approaches that leverage differences in bacterial *k*-mer frequencies to recover the relative abundances of bacteria in complex samples. The setup of the CS problem is similar to our depiction in Figure 3. In Quikr, each column of the sensing matrix Φ is populated with the 6-mer frequency profile of a bacterial species’ 16S gene. Sequence measurements across a whole sample are converted to raw 6-mer frequencies (*y*) from which the sparse combination of species can be recovered using CS with sparsity-based optimization. Quikr was soon followed up with WGSQuikr (110) that leveraged the same core method except with 7-mer analysis of whole-genome shotgun sequencing data. At the time of publication, these techniques achieved competitive accuracy with orders of magnitude improvement in speed over state-of-the-art read-by-read classifiers. However, they were limited to genus-level taxonomic depth and exhibited difficulty in recovering rare organisms.

**MetaPallette** (112) takes a CS-inspired approach similar to WGSQuikr for metagenomic community reconstruction with a few subtle but significant differences. The authors define a matrix *A* created from *k*-mers of database reference genomes, known as the common *k*-mer training matrix. This matrix is analogous to the sensing matrix Φ in CS, but *A* stores pairwise similarities of reference genomes based on shared *k*-mers. *A* is able to be efficiently constructed for long *k*-mers by using bloom count filters. Ultimately, the relative taxa abundances *x* is recovered from the aggregate sample *k*-mer counts *y* by solving *Ax* = *y* for a sparse *x*. While we only discuss a single *A*, *x* and *y* here, MetaPallete in fact creates multiple *A* and *x* for different values of *k* for *k*-mers (30 and 50). The authors also augment *A* with artificial ’hypothetical organisms’ of similar k-mer profiles. The use of long k-mers and the mathematical representation of unknown organisms enables MetaPallette to classify even novel organisms at the strain level.

**MISSION** (113) is a hybrid compressed sensing and hashing-based approach. Specifically, MISSION uses a Count-Sketch data structure and will acquire the *heavy hitters* from the data and apply stochastic gradient descent to update the data structure. The sparsity of the features keeps the top *heavy hitters* while setting the rest to zero. This algorithm was used for metagenomic classification on the dataset from (114) and showed how many features of the data would be adequate relative to performance.

### Metagenome probe design

Metagenomic sequencing has opened the gate for biologists to detect novel or rare organisms in different environments. However, detection with high sensitivity can demand extensive sequencing runtimes to capture novel fragments among the innumerable metagenomic background data (115). To circumvent these challenges, single stranded nucleic acid *probes* can enrich or sense DNA fragments by binding to intended target strands. Many software packages have been developed for designing probes for a specific target genome, but generating probes for metagenomic analysis is difficult because of the uneven and diverse sequences in metagenomic samples. Capturing rare sequences while excluding highly similar sequences is challenging. Therefore, metagenomics requires probe design techniques that scale well with the number of organisms found in samples.

**CATCH** is a newly developed method to design optimal probes for targeted microbial enrichment to facilitate downstream detection in sequencing (116). This approach is particularly important for viral detection in samples with low titers; without probe-based enrichment, low abundance viruses may evade detection. Moreover, CATCH pursues a set of probes that can scalably capture the full diversity of viruses. CATCH first yields a set of candidate probes from the input sequences and then collapses the probes with high similarity using LSH. Specifically, it detects nearly-identical probes through either Hamming distance or MinHash, and then removes the similar candidate probes. To make sure that the final set of probes encapsulates the diversity of the input sequences, CATCH computes the smallest set of probes needed to cover the whole set of target sequences. CATCH treats this as a set cover problem and solves it using the canonical greedy solution (117). Ultimately, thousands of probes are chosen to cover the targets based upon the optimization criteria.

**InSense** While CATCH focuses on probe design for enrichment of target sequences in a complex sample before metagenomic sequencing, applying CS permits another workflow with orders of magnitude fewer probes at the cost of some taxonomic depth. If a sample is known to be $v$-sparse, i.e. contain a subset of $v$ or fewer of the *N* possible microbes, CS can be applied with *M* = *O*($v$log(*N*/$v$)) mismatch-tolerant DNA probes. The sensing matrix Φ is populated by the expected number of binding events between each probe (in rows) and each target organism (in columns). These nonspecific probes can be thought of as directly measuring the abundance of soft-matching *k*-mers. Proof-of-concept work was first explored in a CS microarray (CSM) format (118). The same principle has also been demonstrated for sensing bacterial pathogen genomes at species resolution in bulk solution with less than a dozen fluorescent, random DNA probes (119). Although fewer probes can be resolved in bulk solution compared to a microarray (*M* is limited), such an approach may find applications in rapid infection diagnostics where the species library is constrained to pathogens (*N* is much smaller) and patient samples are very sparse with at most a few unique species (120). Given a set of possible microbes (library), a set of probes, and the simulated hybridization behavior between them, a subset of probes can be selected with the InSense algorithm (73). InSense optimizes for the *incoherence* of Φ, a common quality metric for CS sensing matrices, with a convex relaxation.

This CS approach bypasses sequencing by capturing information directly from probe-target hybridization events, and it will be exciting to see how it performs in real patient and environmental samples. If Φ can be accurately predicted from probe and target sequences, it is plausible that future applications can synergize with sequencing databases by automatically updating Φ based on known trends in microbial evolution. However, nonspecific hybridization mandates a thorough understanding of the library of possible species and perhaps careful sample processing; out-of-library, unexpected nucleic acids that interact with nonspecific probes would corrupt the measurements and downstream sparse recovery.

## DISCUSSION

Despite the nascent state of metagenomic sequencing and analysis, its accelerated adoption has led to both an explosion in available data as well as an ever increasing demand for new data analysis methodologies. In this survey, we have covered what we believe to be a core set of fundamental probabilistic data structures and algorithms that are uniquely positioned to tackle the burgeoning growth of metagenomic data, as well as the added nuances of analyses involving a diverse community contained inside of a metagenome. Despite the relative youth of the field of metagenomics, many fast methods have already emerged that can be used or were designed for this area. For instance, as seen in Table 2, methods like BinDash and Dashing are being developed in an effort to further accelerate sequence similarity estimations beyond the speed of the seminal Mash tool. Similarly, recent advances like BIGSI, RAMBO, and SSBT are opening the door to petabyte-scale sequence searches among vast sequencing datasets.

However, continued breakthroughs are still needed to better handle metagenomic-specific intricacies such as sequencing error, low abundance community members, and uneven coverage. In addition, probabilistic approaches as discussed in this paper generally come with an accompanying set of pros and cons. For instance, most Bloom filter algorithms involve a fundamental trade-off between memory, query cost, and quality. Standard Bloom filters balance the size of the bit array with the possibility of false positives. The tradeoff is implicit for any algorithm using this data structure. The FPR can be reduced by choosing the right number of hash functions, which may increase query time, or by making assumptions about the input data, as with *k*-mer Bloom filters. Cascading Bloom filters provide an alternative way to trade query time and memory for FPR at the expense of a more complex hierarchical structure.

Additionally, CS approaches come with their own set of tradeoffs. While CS confers measurement efficiency for cost and time savings, it is inherently database-dependent. For instance, in some of the applications we discussed, the sensing matrix Φ was precomputed by leveraging a sequence database (sequences at a specified position, *k*-mer frequencies, response to a set of probes etc.). Similarly, the discovery of sparse representations requires a training set of signals. This requirement for a dataset becomes limiting in chaotic applications such as the identification of rapidly evolving organisms either through vertical or horizontal gene transfer. Such novel differences that real-world samples may exhibit would likely be treated as noise in sparse recovery and ignored until the database is updated. CS is therefore likely limited to applications that exhibit an acceptable level of stability in the dataset. More generally, while the CS technique is provably robust to errors (noise) in the low-dimensional measurements *y*, any errors in the signal *x* are amplified by the factor *N*/*M* (121). In metagenomics, measurement noise may be attributed to whether an expected nucleic acid fragment in the sample generates a read during sequencing, and signal noise could be the result of unforeseen mutations or contamination. In applications featuring significant signal noise, the ratio *N*/*M* controls the tradeoff between the efficiency of the measurement process and signal-to-noise ratio degradation.

In addition to all of the considerations directly involved in the inner workings of the discussed methods, there are many considerations surrounding these methods that can also greatly affect both their accuracy and scale. While we have discussed various tradeoffs involved in probabilistic approaches, many of these tradeoffs involve carefully selected hyper parameters. To a non expert user of the methods, it may not be obvious how to set the various parameters for each method, and even advanced users may struggle to find the truly optimal parameter settings derived from underlying theory. Another consideration is in the modeling of processes such as natural genome evolution. Many *k*-mer based approaches and hashing techniques are initially developed in a way that is blind to underlying biological processes such as evolutionary drift which gradually introduces point mutations, insertions, and deletions into closely related genomes that otherwise might be identical. Conversely, phylogenetic methods which explicitly model events like drift and recombination have been slow to incorporate recent advances discussed in this survey. Considerations can also be given to the actual data collection procedures, such as how the DNA sequencing is performed. One new advance on the sequencing side of metagenomics is the concept of genome skimming (122), which is a technique to lightly sequence metagenomic samples. Similarly, Metabarcoding (107) or amplicon sequencing can reduce metagenomic data by only sequencing a small set of amplified regions, potentially speeding up and simplifying downstream analyses.

A final consideration surrounding newer methodologies is that of the sequence databases that nearly all metagenomics tools rely on for sequence classification. While recent advances in probabilistic data structures and algorithms may drastically shrink computational requirements, these speedups can be easily offset and even outpaced by exponential growth in sequence databases that these algorithms must interact with. New methods should also seek to overcome challenges such as database quality issues such as misassembled or mislabelled genomes or sets of reads. Following methodologies such as simple uniform random downsampling and more intelligent downsampling like Diginorm (123), recent advances like the RACE method (66) attempt to address the need to shrink databases and remove contaminants and error, while preserving biologically important characteristics like diversity.
